# Tanshinone IIA promotes the differentiation of bone marrow mesenchymal stem cells into neuronal-like cells in a spinal cord injury model

**DOI:** 10.1186/s12967-018-1571-y

**Published:** 2018-07-13

**Authors:** Xue-Mei Zhang, Jiao Ma, Yang Sun, Bing-Qian Yu, Zhuo-Min Jiao, Duo Wang, Mei-Yu Yu, Jin-Yue Li, Jin Fu

**Affiliations:** Department of Neurology, The Second Affiliated Hospital of Harbin Medical University, No. 246 XueFu Road, Nangang District, Harbin, 150001 People’s Republic of China

**Keywords:** Spinal cord injury, Tanshinone IIA, Bone marrow mesenchymal stem cells, Differentiation

## Abstract

**Background:**

Spinal cord injury (SCI) is one of the most severe central nervous system injuries. Currently, transplanting bone marrow mesenchymal stem cells (BMSCs) is considered a therapeutic option for SCI. Tanshinone IIA (TIIA) is one of the extracts obtained from *Salvia miltiorrhiza Bunge*, which has been shown to have some protective effects against SCI. The present research was aimed to explore whether TIIA would influence the fate of transplanted BMSCs in a rat model of SCI, especially with regard to their differentiation into neuronal cells.

**Methods:**

Bone marrow mesenchymal stem cells were obtained from immature rats and identified using flow cytometry. After SCI, 1.0 × 10^7^ cells labeled with PKH67 were transfused into the injured spinal cord. TIIA was first injected into the tail vein (30 mg/kg) 1 h before surgery. From day 1 to day 7 post-SCI, TIIA was injected (20 mg/kg) per day at the same time. Recovery of locomotor function and histological regeneration of the spinal cord were compared among the groups, with the differentiation and distribution of BMSCs determined anatomically and biochemically by the expression of neural cell markers.

**Results:**

Locomotor assessments showed that the rats in the BMSCs + TIIA group exhibited higher scores (19.33 ± 0.58) than those in the other groups (13.67 ± 1.53, 17.67 ± 0.58, 18.00 ± 1.73). The area of the cavity in the BMSCs + TIIA rats was smaller than that in the other groups (1.30 ± 0.56, 10.39 ± 1.59, 6.84 ± 1.18, 4.36 ± 0.69). Co-expression of glial fibrillary acid protein was observed in transplanted BMSCs, with a reduced rate in the BMSCs + TIIA group relative to that in the SCI group. In contrast, the expression levels of Nestin, neuron-specific nuclear protein (NeuN) and neurofilament protein 200 (NF200) were greatest in the transplanted cells in the BMSCs + TIIA group.

**Conclusions:**

Tanshinone IIA treatment enhances the therapeutic effects of BMSC transplant on SCI, likely by promoting the differentiation of neuronal cells.

## Background

Spinal cord injury (SCI) is one of the most severe central nervous system (CNS) lesions that affects a patient’s physical and mental health, has a heavy economic burden on society and detrimentally affects the patient’s family [1]. There currently is no effective treatment strategy for SCI in clinical practice, with treatment opinions entailing the use of high dose methylprednisolone, surgical interventions to stabilize and decompress the spinal cord, and rehabilitative care. However, cellular, molecular, or rehabilitative training with combinatorial therapies are beginning to show promising results in animal models [2]. Among the potential drugs that may be helpful for SCI is *Salvia miltiorrhiza Bunge (SMB),* a traditional Chinese herb that is currently used for the treatment of various diseases, in particular cardiovascular and cerebrovascular cases [3, 4]. Tanshinone I, tanshinone IIA (TIIA) and cryptotanshinone are the three major bioactive diterpenoid quinones isolated from *SMB* [5]. TIIA is the most abundant diterpene quinone, which has potential protective effects against atherosclerosis, cardiac hypertrophy, cardiac fibrosis, also can attenuate oxidative stress induced apoptosis [6]. Thus it recently has been used in treating cardiovascular diseases, specifically hypertension and myocardial infarction [3]. It also appears to have a protective effect against SCI by inhibiting proinflammatory processes and SCI-induced apoptosis [7, 8].

With the development of cell and molecular biology, the transplantation of stem cells has been considered a promising therapeutic treatment [9]. Recently, bone marrow mesenchymal stem cells (BMSCs) have received much attention since they are easily isolated and cultured, and they exhibit a favorable proliferative profile, an ability for polydirectional differentiation and a reduced immunological reaction. In view of these prospects, BMSCs have been used as a therapy for orthopedic, cardiovascular and neurological diseases [10–13]. Our previous data demonstrate that BMSCs can improve function in a cerebral ischemia model [14], and BMSCs have been used in the clinic for the treatment of SCI [15]. In recent years, scientists have proven that TIIA could increase BMSCs migration into the infarct region in a myocardial ischemia model [16, 17]. However, there is no data on the combined therapeutic effect of TIIA treatment with stem cells transplantation on SCI. The aim of the current study was to investigate whether TIIA could promote differentiation of BMSCs into neurocyte-like cells in an SCI model and subsequently improve behavioral outcome and morphology.

## Methods

The Medical Ethics Review Committee of Harbin Medical University approved all experiments in accordance with the Detailed Rules for Medical Research Purpose issued by the Ministry of Health, China (No. HMUIRB20150029). The primary antibodies that we used were purchased from Abcam (USA) (Table 1).

**Table 1:** Primary antibodies

Antibody	Cat. No.	Source	Concentration
GFAP	ab33922	Rabbit monoclonal antibody	IHC (1:400) IF (1:200) WB (1:2000)
Nestin	ab11306	Mouse monoclonal antibody	IHC (1:200) IF (1:200) WB (1:2000)
NeuN	ab10224	Mouse monoclonal antibody	IHC (1:200) IF (1:200) WB (1:10,000)
NF200	ab82259	Mouse monoclonal antibody	IHC (1:100) IF (1:200) WB (1:10,000)

*IHC* immunohistochemistry, *IF* immunofluorescence, *WB* western blotting

### Animal preparation

Sprague-Dawley (SD) female adult rats (7 weeks old, weighing 200–220 g) were purchased from the animal center of the Second Affiliated Hospital of Harbin Medical University. The rats were housed at room temperature (25 °C) and were provided a standard laboratory diet and water ad libitum.

### Spinal cord injury

Rats were anesthetized by intraperitoneal injection of 10% chloral hydrate (3 mL/kg). Under aseptic conditions, hair was removed from the T8–T11 region of the back, the skin was sterilized with antiseptic betadine, and then a 2-cm midline incision was made. The spinal cord was exposed by laminectomy at the T9–T10 level, without damaging the dura mater. An SCI was generated by using an aneurysm clip exerting a 0.6-N closing force on the spinal cord for 1 min [18]. Subsequently, muscle and skin were sutured with 3-0 Vicryl suture. Post-operatively, gentamicin was added into the water at a dose of 500 g/L for 7 days to prevent infection. Body temperature was kept at 37 °C by using a warm blanket during surgery and the post-operative recovery period. Two rats without hindlimb paralysis post-surgery were excluded from this study. Urine was manually cleared from rats post-operatively twice daily (i.e., for 7–14 days) by pressing the bladder until the rats could urinate by themselves.

### Study design

A total of 56 rats were used in this study, eight were sacrificed for gross findings and electron microscopy analysis. The other 48 SCI rats were randomly assigned to four groups. (1) Group A was the control group (SCI only, *n *= 12); (2) Group B was the TIIA group (injection of TIIA, *n *= 12); (3) Group C was the BMSCs group (transplantation of BMSCs, *n *= 12); (4) Group D was the BMSCs + TIIA group (transplantation of BMSCs and injection of TIIA, *n *= 12; Sup. 1). The chemically pure TIIA were purchased from Klamar reagent company (Cas. No. 568729). The TIIA’s purity is more than 98% and has no immunogenicity, so it is used safely in our experiments. TIIA was initially injected 1 h before surgery via the tail vein (30 mg/kg) and then on days 1–7 post-SCI, and TIIA (20 mg/kg) was administered once a day at the same time [16, 19]. BMSCs (1.0 mL containing 1.0 × 10^7^ cells/mL) were intravenously transplanted 3 h after SCI. Before transplantation, BMSCs were labeled with PKH67 to assess their localization later. Control rats received the same volume of a 0.9% sterile saline solution via the tail vein. An open field locomotor assessment was performed on all animals using the Basso, Beattie, Bresnahan (BBB) scale at 1, 7, 14, 21, and 28 days post-surgery. On the first day post-surgery, one SCI rat and one normal control rat were sacrificed and the injured region of spinal cord was collected for gross findings. At 2 days post-SCI, the spinal cords from three SCI rats and three normal rats were collected for electron microscopy analysis. The remaining animals were sacrificed at 28 days post-SCI for H&E (hematoxylin and eosin) staining, immunohistochemistry, triple immunofluorescent staining, western blotting and real-time PCR analysis.

### Isolation, culture and differentiation of BMSCs

Rat BMSCs were isolated using a density gradient centrifugation method and were then cultured according to a protocol previously described [20]. Eight SD male rats (4 weeks old) were deeply anesthetized with an overdose of 10% chloral hydrate. Bone marrow was flushed out from the femoral and shin bones using 4.0 mL of Dulbecco’s modified Eagle’s medium F12 (DMEM/F12, HyClone) under aseptic conditions. Bone marrow was then added to an equal volume of rat lymphocyte separation medium (density: 1.083 g/mL, Solarbio, China) in a 15.0-mL plastic centrifuge tube and centrifuged at 2500 rpm for 30 min (centrifugal radius = 20 cm) with bone marrow mononuclear cells harvested from the second layer (from top to bottom). The collected cells were washed in DMEM/F12 and resuspended in T25 culture flasks with DMEM/F12 supplemented with 10% heat-inactivated fetal bovine serum (FBS; Sciencell, USA), 100 U/mL penicillin and 0.1 mg/mL streptomycin (Beyotime, China). The bone marrow mononuclear cells were then plated at a density of 5.0 × 10^6^ cells per flask and incubated in a humidified atmosphere of 5% CO_2_/95% O_2_ at 37 °C. Three days later, the non-adherent cells were removed and then added to fresh culture medium; the medium was changed every 2–3 days. When cells reached 80–90% confluence, they were digested with 0.25% trypsin and 0.02% EDTA (Beyotime, China) and subcultured. Cells were cultured for three passages for identification, labeling and transplantation. Adipogenesis was induced by using the OriCell™ adipogenic differentiation kit (Cyagen, Guangzhou, China). The OriCell™ osteogenesis differentiation kit (Cyagen, Guangzhou, China) was used to induce osteogenic differentiation. The capacity of the BMSCs for differentiation to adipogenic and osteogenic cells was assessed as described previously [21].

### Identification and labeling of BMSCs

Flow cytometric analysis was performed to identify the cellular surface phenotype of cultured cells. BMSCs were treated with trypsin–EDTA solution and then washed three times with PBS containing 10% bovine serum albumin (BSA; Beyotime). The cells then were added and incubated with primary monoclonal rat antibodies including phycoerythrin (PE)-conjugated CD29, allophycocyanin (APC)-conjugated CD90, fluorescein isothiocyanate (FITC)-conjugated CD45, purified CD73, purified CD34 and CD44 for 30 min at 4 °C. The secondary antibodies included PE-conjugated rat anti-mouse IgG1 and FITC-conjugated goat anti-rabbit IgG H&L. The isotype-matched controls included mouse G1-PE, mouse G1-APC, mouse G1-FITC, hamster IgM-PE, and rabbit IgG (Abcam, USA). Labeled cells were analyzed by a FACS Calibur flow cytometer (Becton–Dickinson) using Cell Quest software.

Before transplantation, the BMSCs were labeled with the fluorescent membrane dye PKH67 (green fluorescence dye, Sigma). PKH67 is recommended for short-to-medium term in vivo studies that require a green cell linker dye, as well as in vitro cytotoxicity, proliferation or other co-culture assays [22, 23]. After surgery, 1.0 × 10^7^ cells labeled with PKH67 (the BMSCs and BMSCs + TIIA groups) were resuspended in 1.0 mL of 0.9% sterile saline solution and injected slowly through the tail vein over 5 min [24].

### Locomotor activity test

The recovery of locomotor function was assessed using the BBB scale [25]. The BBB 21-point open field locomotor rating scale (0 = complete paralysis, 21 = normal gait) was performed at 1, 7, 14, 21, and 28 days after surgery to evaluate hindlimb movement. Animals were placed individually in a circular enclosure and allowed to move freely for 5 min. Two independent examiners who were blinded to the treatment groups monitored the animals’ behaviors, recorded their scores and calculated the average score.

### Histopathology and electron microscopy analysis

**Fig. 1:**
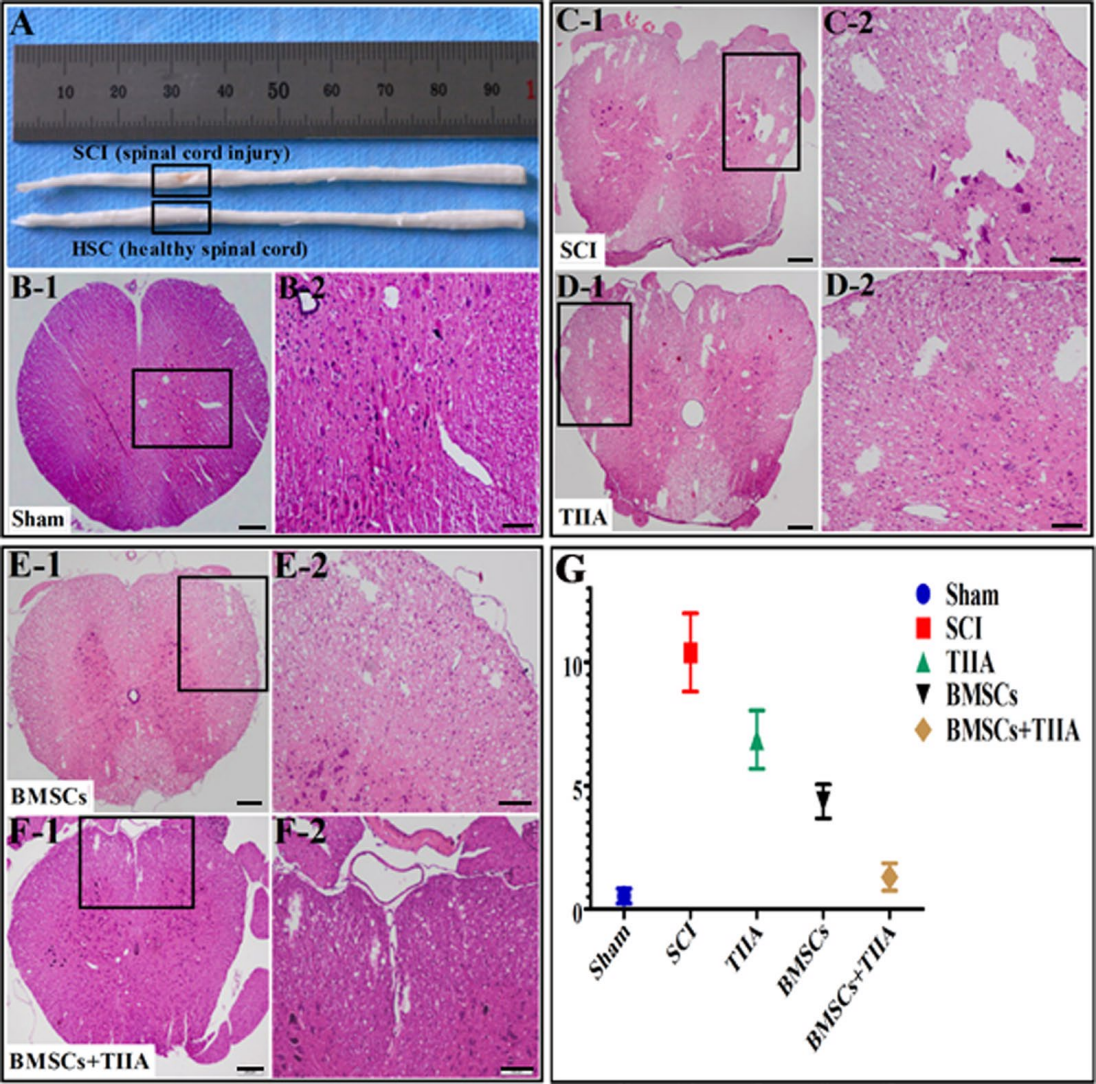
Gross findings, H&E images of injured spinal cord lesions and image analysis. **A** Gross findings at 1 day post-SCI and a healthy control. The yellowish connective tissue (inside rectangle) was the site of SCI. It (rectangle) showed damage and deflection compared to the healthy spinal cord. **B** H&E images of normal spinal cord. **C**–**F** H&E images of injured spinal cord (28 days post-SCI). Damage to the spinal cord was associated with the presence of a cavity and parenchymal necrosis at 28 days after SCI. **G** Image analysis of cavity formation at the epicenter of the SCI. There was no significant difference between the sham and BMSCs + TIIA groups. The cystic area of the SCI group was the largest and there were differences between these groups (SCI vs TIIA, SCI vs BMSCs, SCI vs BMSCs + TIIA, TIIA vs BMSCs, TIIA vs BMSCs + TIIA, BMSCs vs BMSCs + TIIA,***P < 0.001). Scale bar: 200 μm (**C-1, D-1, E-1** and **F-1**); 100 μm (**C-2**, **D-2**, **E-2** and **F-2**)

On the first day post-surgery, one SCI rat and one normal rat were deeply anesthetized with 10% chloral hydrate (6.0 mL/kg, i.p.) and intracardially perfused with 0.9% saline solution followed by 4% paraformaldehyde in 0.01 M PBS (pH 7.4, PBS). The spinal cord was extracted, and a picture was taken of the center of the lesion (Nikon, Japan; Fig. 1A). After these procedures, tissue located at 1.0 cm rostral to 1.0 cm caudal of the injury epicenter was fixed in 10% formalin overnight, and embedded into paraffin. Tissue blocks were divided into 4.0-μm thick slices. A total of three pieces were taken from each of the ten pieces and stained with H&E to examine the edema and defect. The volumes of cavities and total spinal cord in each slide were calculated using ImageJ software (version 1.46; National Institutes of Health, USA).

The other three SCI rats and three normal rats were sacrificed at 2 days post-SCI, and the spinal cord sections 1.0 cm rostral to 1.0 cm caudal to the injury site were removed and fixed in 3.6% glutaraldehyde containing 0.1 M phosphate buffer for 15 min. A 1-mm slice was cut transversely at the lesion epicenter, fixed in buffered osmium tetroxide, dehydrated in cold graded ethanol and embedded in Epon Araldite (Electron Microscopy Sciences, Fort Washington, PA). Sagittal sections (1.0 μm) were stained with 1% toluidine blue. A plastic-embedded cross-section of spinal cord was divided into thirds, and these areas were carefully mapped before thin sectioning. Thin sections were stained with uranyl acetate and lead citrate and examined with an electron microscope (JEM-1400, JEOL Ltd., Tokyo, Japan) operating at 80 kV [26].

### Immunohistochemistry and immunofluorescence

Rats were sacrificed was at 28 days (*n *= 24) post-surgery. The tissues for immunofluorescence were immersed in 30% sucrose until they sank. The spinal cord was cut transversely using a cryostat at a thickness of 20.0 μm with sections mounted on frosted glass slides and stored at − 20 °C until use. Primary monoclonal rat antibodies included rabbit anti-GFAP, mouse anti-Nestin, mouse anti-NeuN, and mouse anti-NF200.

For immunohistochemistry using the avidin–biotin-complex (ABC) method, sections were first treated with 3% H_2_O_2_ for 30 min; blocked in PBS containing 10% goat serum, 0.2% Triton X-100 and 5% BSA for 1 h at room temperature; and then were reacted with primary antibodies (for GFAP, Nestin, NeuN, and NF200) overnight. The next day, sections were incubated with biotinylated pan-specific secondary antibodies (goat anti-mouse IgG, goat anti-rabbit IgG, Boster, China) at 1:200 for 2 h, followed by the ABC step (1:400, Boster, China) for 1 h. Immunoreactivity was visualized using 0.05% 3,3′-diaminobenzidine (DAB) and 0.003% H_2_O_2_.

To assess the survival, migration, and proliferation of PKH67-labeled BMSCs, an immunofluorescence triple staining protocol was performed using a previously described procedure. Briefly, after washing sections with PBS, they were incubated in Alexa-Flour 594 conjugated goat anti-rabbit and mouse IgG (1:100, MultiSciences, China) for 1 h. Finally, sections were counterstained with 4′, 6′-diamino-2- phenylindole (DAPI) dihydrochloride (1:100, Boster, China) and mounted with anti-fading medium before images were acquired.

Signals were detected and imaged using an Olympus microscope (BX41, Olympus, Japan) and confocal fluorescence microscope (FV-1000 spectral, Olympus, Japan) at 535 nm/565 nm (rhodamine, red) and 470 nm/505 nm (FITC, green) using a digital camera. The numbers of positive cells were determined by averaging the numbers of cells counted in six randomly selected areas of three non-consecutive sections in each group and calculated using the ImageJ and FLUOVIEW in the confocal microscope system (×200, ×400).

### Western blotting

Animals were deeply anesthetized with an overdose of 10% chloral hydrate at 28 days post-surgery. The lesioned spinal cords (2.0 cm in length) were extracted and stored at − 80 °C. The tissue was then homogenized on ice in radioimmunoprecipitation assay (RIPA) buffer (150 mM NaCl, 50 mM Tris–HCl pH 7.4, 2 mM EDTA, 1% NP-40, 0.1% Triton X-100, 0.1% sodium dodecyl sulfate (SDS), 1 mM Na_3_VO_4_, 1 mM sodium deoxycholate, 1 mM PMSF, 10 mg/mL aprotinin, and 5 mg/mL leupeptin) and centrifuged at 12,000 rpm for 20 min at 4 °C. The supernatant (50 μg of total protein) was separated on 10% SDS gels (Beyotime) and transferred to polyvinylidene fluoride (PVDF) membranes (Millipore, Bedford, MA, USA). After blocking with 10% non-fat milk in TBST (25 mM Tris–HCl, 0.15 mM NaCl, 0.1% Tween 20) for 1 h, the PVDF membranes were incubated at 4 °C overnight with primary antibodies: rabbit anti-GFAP (1:2000, Abcam, USA), mouse anti-Nestin (1:1000, Abcam, USA), mouse anti-NeuN (1:5000, Abcam, USA), mouse anti-NF200 (1:2000, Abcam, USA) and β-actin (1:500, ZSGB Biological, China). After washing with PBS containing 0.1% Tween 20, the membranes were incubated with appropriate secondary antibodies: horseradish peroxidase-labeled goat anti-rabbit IgG and goat anti-mouse IgG (1:5000, ZSGB Biological, China) for 2 h. Finally, membranes were washed again, and the bands were detected with an enhanced chemiluminescence (ECL) detection kit (Beyotime, China) and exposed to X-ray films. Western blotting bands were quantified using Quantity One software.

### Real-time PCR analysis

Gene expression in the injured spinal cord was examined using real-time PCR. Briefly, animals were deeply anesthetized with an overdose of 10% chloral hydrate on day 28 post-surgery (*n *= 12), sacrificed and their tissue (1.0 cm rostral to 1.0 cm caudal of the injury epicenter) was collected. Total RNA was extracted using an illustra RNAspin Mini isolation RNA Kit according to the manufacture’s protocol (Sangon Biotech, China). A total of 1 μg of RNA was then incubated with a reverse transcription mixture at 42 °C for 50 min. PCR amplification was then performed with 200 nM primers for rat GFAP, Nestin, NeuN, and NF200 (Table 2). PCR conditions entailed 95 °C for 15 s, followed by 30 cycles at 95 °C for 5 s, and finally 60 °C for 30 s. The experiments were performed three times, results were averaged, and each value was normalized to GAPDH mRNA levels. The relative quantification level of gene expression for the control group was determined using the 2^−△△CT^ method.

**Table 2:** Primers used for PCR analysis

Gene	Primer sequence (5′-3′)	
Rat	GFAP	F: CGAAGAAAACCGCATCACCA
		R: CATCCTTGTGCTCCTGCTTC
Rat	Nestin	F: GGCAAATCTGGGAACTGGTA
		R: GACATCAGTGGCTCCTCTCC
Rat	NeuN	F: ACCCTCCTCCACCTCAGAAT
		R: GTCTGTGCTGCTTCATCTGC
Rat	NF200	F: GAGTGGTTCCGAGTGAGATTC
		R: TGCTTTTCAGTGCCTCCAAC
Rat	GAPDH	F: AAGAAGGTGGTGAAGCAGGC
		R: TCCACCACCCTGTTGCTGTA

### Statistical analysis

The data are presented as the means ± SDs and were analyzed using GraphPad Prism 5.0 software (GraphPad, La Jolla, CA, USA) and ImageJ. A one-way analysis of variance (ANOVA) and Student’s *t* test (paired) were used to compare multiple groups and two groups, respectively. Analysis of the BBB scale data was performed by two-way ANOVA of repeated measures. A value of *P *< 0.05 was used to deem statistical significance.

## Results

### Histological and microscopic changes after SCI

To compare the extent of SCI, we assessed the histological changes at the epicenter of the lesion using gross examination at 1 day after SCI and microscopic examination via light microscopy at 28 days post-surgery. Compared with the normal non-lesioned spinal cord tissue, the injured cord exhibited tissue with a mechanical insult that included edema and loose tissue structures (Fig. 1A). At 28 days after SCI, the cavity formations were examined in all groups (Fig. 1C1–F2). The percentage of cavity formations areas in the transverse section at the epicenter of the injured spinal cord in the sham, SCI, TIIA, BMSCs, and BMSCs + TIIA groups were 0.54 ± 0.30, 10.39 ± 1.59, 6.84 ± 1.18, 4.36 ± 0.69, and 1.30 ± 0.56, respectively. There was no significant difference between the sham and BMSCs + TIIA groups, while there were significant differences among the other groups (i.e., SCI vs TIIA, SCI vs BMSCs, SCI vs BMSCs + TIIA, TIIA vs BMSCs, TIIA vs BMSCs + TIIA, BMSCs vs BMSCs + TIIA, ***P < 0.001; Fig. 1g).

**Fig. 2:**
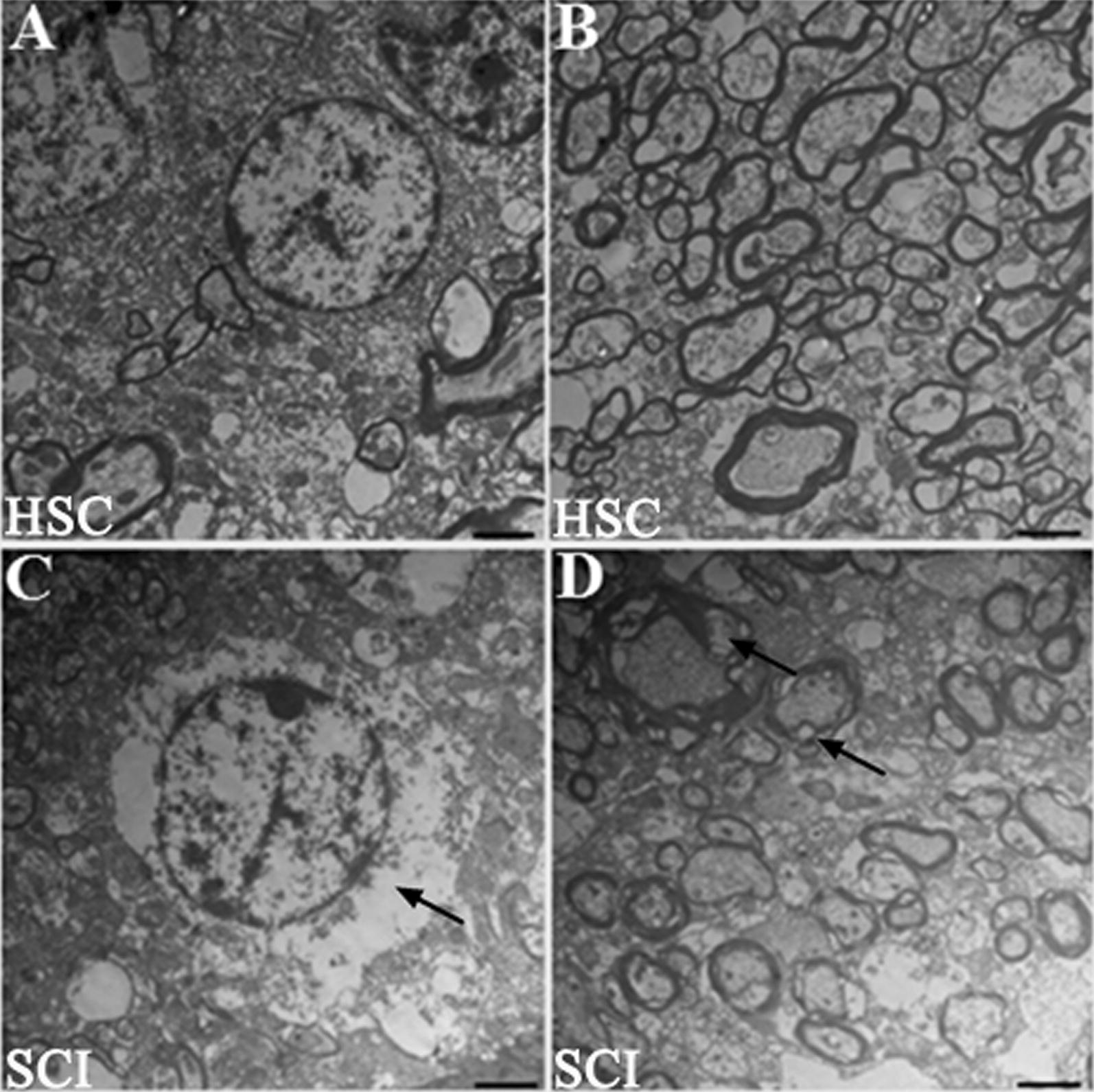
Electron microscopy of neurons and the myelin sheath at the epicenter of healthy and injured spinal cord (HSC and SCI). **A**, **C** The normal (**A**) and injured (**C**) neurons in the spinal cord; there was perineuronal edema (arrow). **B**, **D** The normal (**B**) and injured (**D**) myelin sheath, there was edema (arrows). Scale bar, 2 μm

Compared to those in the normal spinal cord tissue, the neurons and myelin sheath in the ultrathin sections at the epicenter of the injured spinal cord tissue revealed perineuronal and myelin sheath edema (Fig. 2, arrows). The macrophages were rare at 2 days in these sections.

### Characteristics of cultured BMSCs

**Fig. 3:**
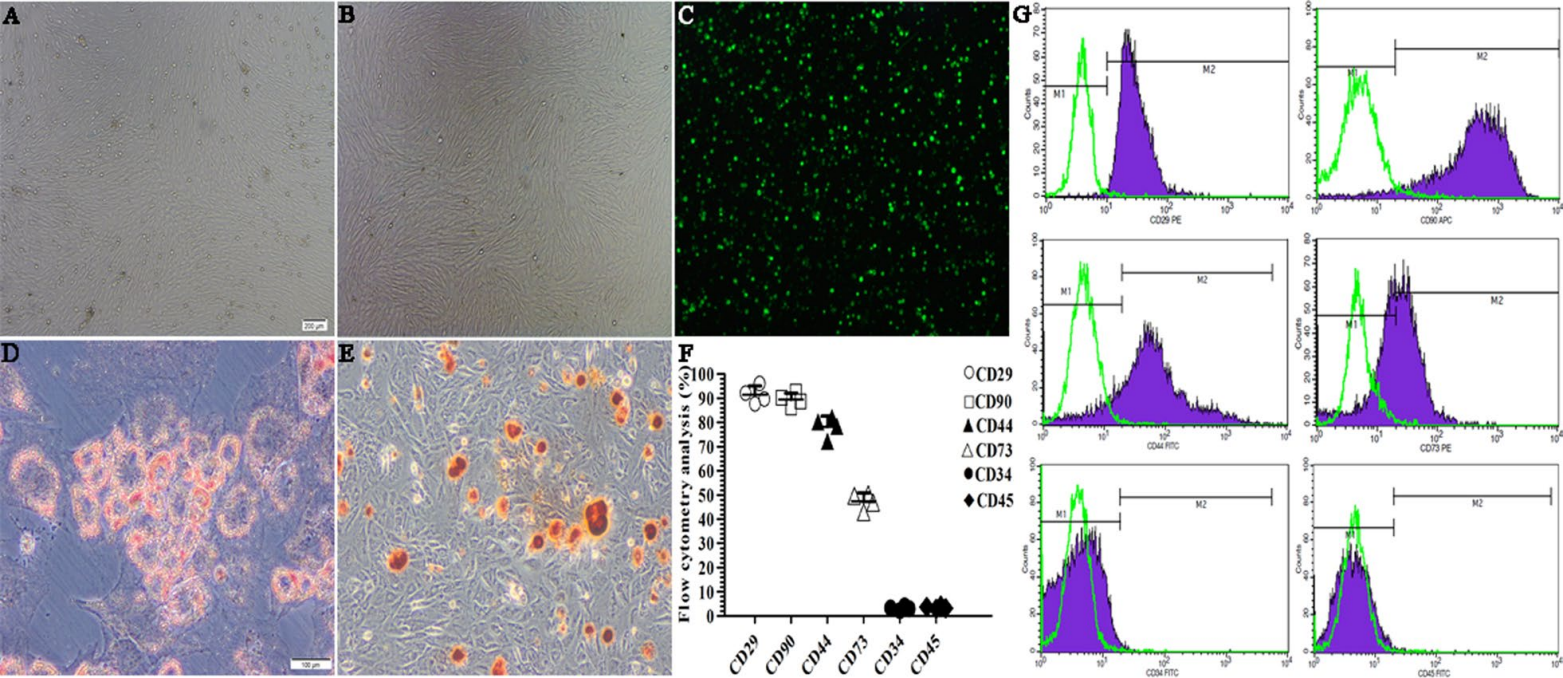
Morphology, identification of PKH67-labeled BMSCs, characterization and FACS analysis of undifferentiated BMSC surface markers. **A**, **B** The morphology of BMSCs at passages 0 (**A**) and 3 (**B**). **C** Before transplantation, cells were marked with the fluorescent membrane-intercalated dye PKH67. **D**, **E** The BMSCs differentiated into the adipogenic and osteogenic lineages. **F**, **G** Flow cytometry analysis of the immune phenotype of cultured cells. The cells were CD29(+), CD90(+), CD45(−), CD34(−), with partial expression of CD73 and CD44. These data indicated that they were of mesenchymal stem cell lineage. Values are the means ± SDs. Scale bar, 200 μm (**A**– **C**), 100 μm (**D** and **E**)

The morphological features of cultured BMSCs at passages 0 and 3 were examined (Fig. 3A, B). At passage 0, the phenotype of the cells was heterogeneous, but at passage 3, the cells had homogeneous spindle-shaped, fibroblast-like morphology. Before transplantation, the BMSCs were labeled with PKH67 and observed under a microscope (Fig. 3C). The BMSCs can successfully differentiated into adipocytes and osteoblasts by Oil Red O staining and alizarin red staining, respectively (Fig. 3D, E). Flow cytometry analysis of the passage 3 cultured BMSCs was performed to confirm immune phenotypes. As shown in Fig. 3F and G, the cells were positive (+) for CD29 and CD90 (91.39 ± 3.64% and 89.40 ± 2.71%, respectively) but negative (−) for CD34 and CD45 (3.44 ± 0.55% and 3.16 ± 0.78%, respectively). Partial expression of CD73 and CD44 cell surface markers was also noted (47.43 ± 3.38% and 78.22 ± 4.30%, respectively). These results indicated that the cell surface phenotype was characteristic of BMSCs and are consistent with previous studies [27, 28].

### Engraftment and differentiation of BMSCs in the injured spinal cord

**Fig. 4:**
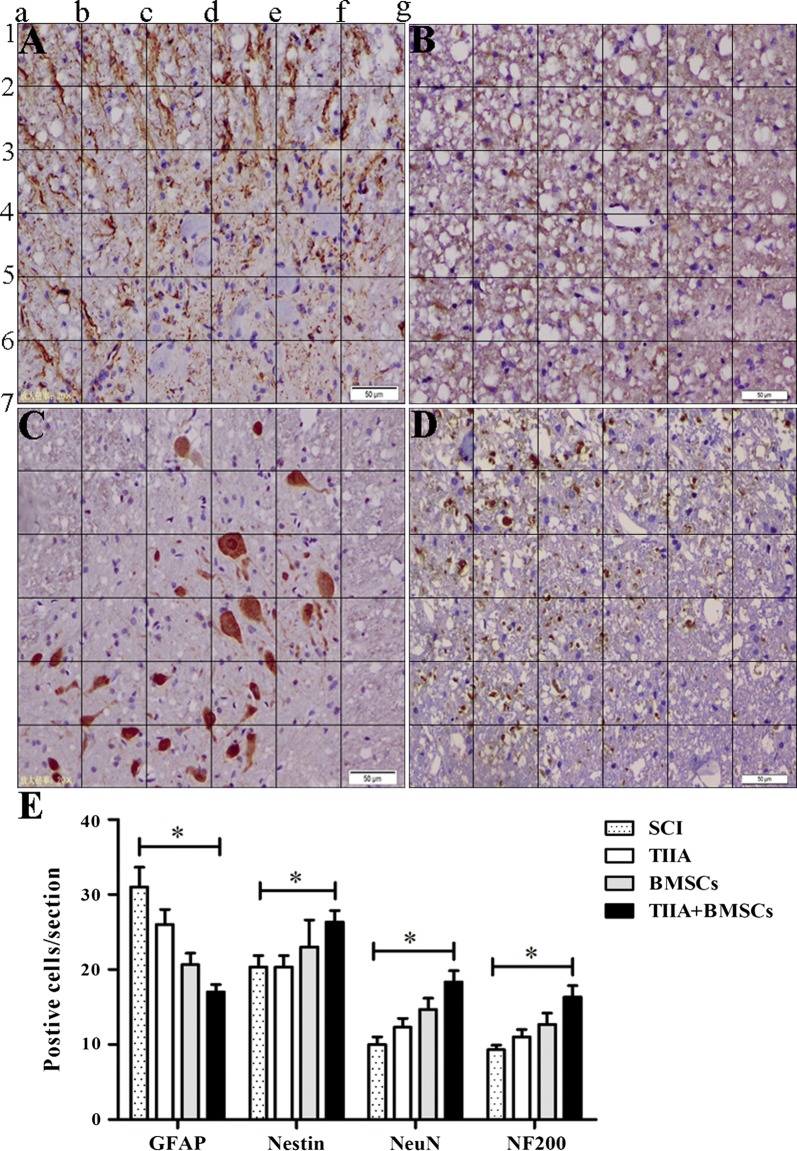
Immunohistochemical staining results and images analysis. **A**–**D** GFAP(+), Nestin(+), NeuN(+), and NF200(+) cells were counted in the vicinity of the spinal cord lesion for each group at 28 days post-injury. **E** The image analysis showed that there were significantly more GFAP(+) cells in the SCI group compared to those in the other groups. In the BMSCs + TIIA group, strong immunohistochemical staining for Nestin, NeuN and NF200 was observed, but no significant difference was noted between the TIIA and BMSCs groups. Images were taken from the BMSCs only group. Values are mean ± SDs (*n *= 3 per group). *P < 0.05, **P < 0.01 versus the SCI group. Scale bar, 50 μm

Immunohistochemical analysis was performed at 28 days post-surgery. As shown in Fig. 4A–D, in the SCI lesion of BMSCs + TIIA group, cells exhibited colocalization of GFAP and neuronal cell markers. However, the number of cells that showed colocalization with GFAP was significantly higher in the SCI group than that in the other groups (*n *= 3, *P < 0.05, **P < 0.01), while the number of cells exhibiting colocalization with Nestin, NeuN, or NF200 was the highest in the BMSCs + TIIA group (*P < 0.05). However, no significant difference was noted in the number of colocalized cells between the TIIA and BMSCs groups (*P *> 0.05; Fig. 4E).

**Fig. 5:**
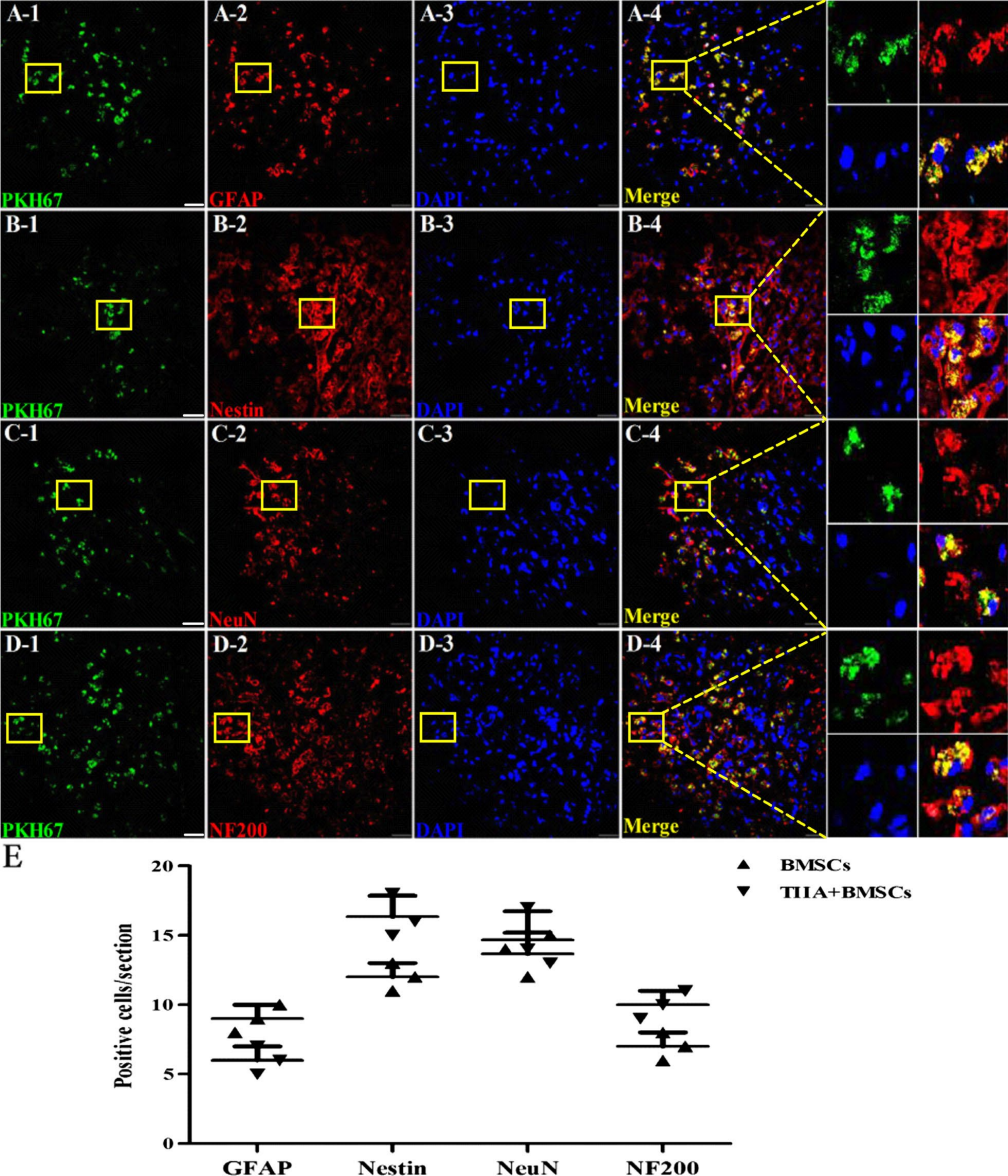
PKH67(+) cells were mainly found at the site of injury and exhibited varying colocalization with glial and neuronal cell markers. **A1**–**4** Astrocytic differentiation of the transplanted BMSCs. **B1**–**D4** Neuronal differentiation of the transplanted BMSCs. Rectangles indicated the colocalization of PKH67 with GFAP, Nestin, NeuN, or NF200 (*n *= 3, three sections and six fields per section). **E** Image analysis showed that the number of transplanted BMSCs expressing Nestin, or NF200 was higher in the TIIA + BMSCs group than that in the BMSCs group (*P *< 0.05), while GFAP expression was higher in the BMSCs group (*P *< 0.05). There was no significant difference of in the number of NeuN(+) cells between the two groups (*P *> 0.05). Images are taken from the BMSCs only group. Data are means ± SDs. Scale bar, 50 μm

Immunofluorescence analysis was performed at 28 days post-transplantation, and the transplanted BMSCs were centralized in the epicenter of the injured spinal cord in BMSCs + TIIA group (Fig. 5A–D). The number of cells exhibiting colocalization of PKH67 and GFAP was higher in the BMSCs group than that in the BMSCs + TIIA group (*n *= 3, *P *< 0.05). In contrast, the BMSCs + TIIA group exhibited more colocalization of Nestin, NeuN, or NF200 with PKH67-labeled cells than that observed in the BMSCs group (*n *= 3, *P *< 0.05; Fig. 5E).

### Protein levels of glial and neuronal cell markers

**Fig. 6:**
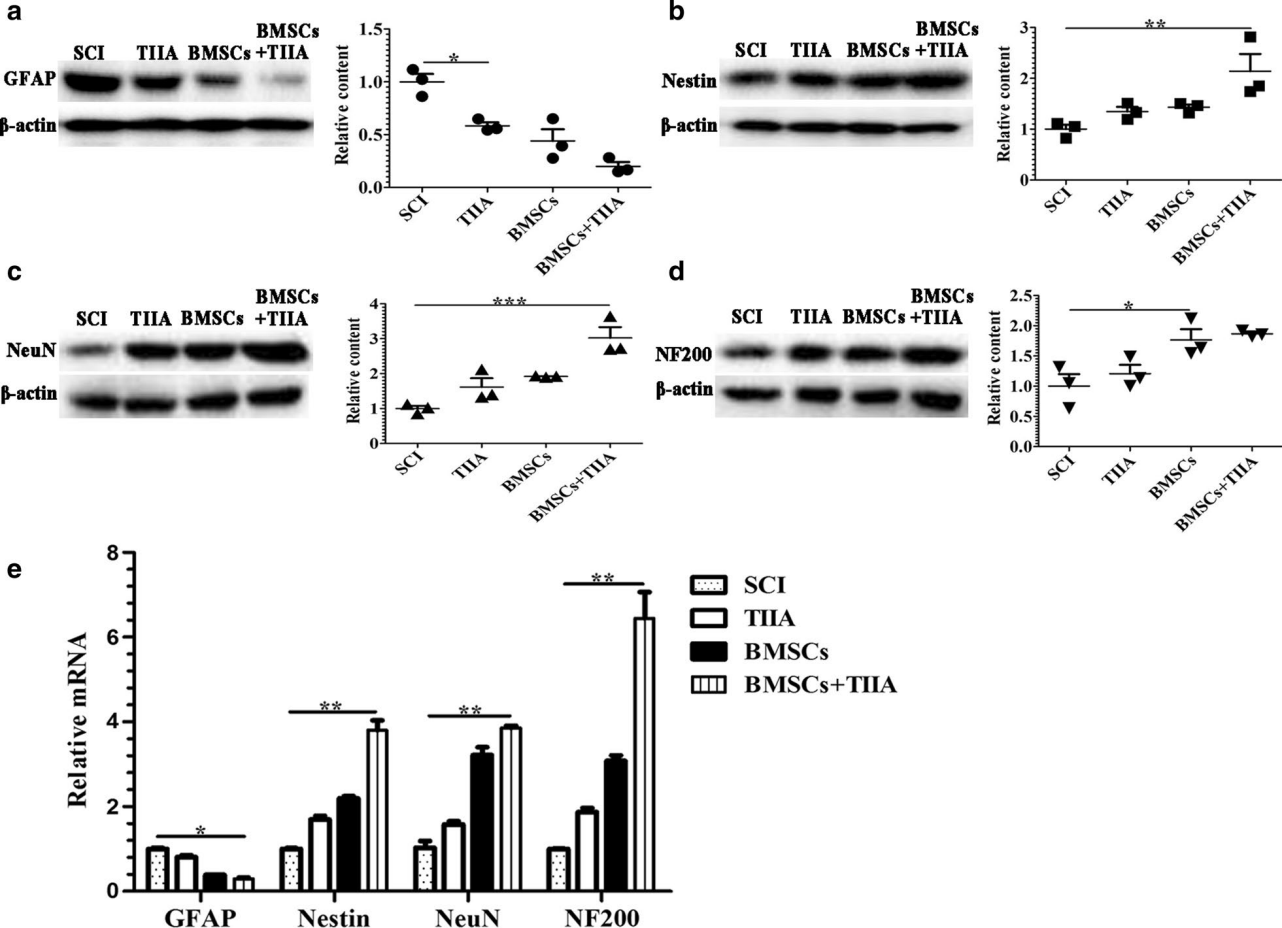
Western blotting and real-time PCR for gene expression of glial and neuronal cell markers. The protein and mRNA expression levels of GFAP, Nestin, NeuN, and NF200 were detected in all groups at 28 days post-SCI. **a** The level of GFAP expression in the SCI group was higher compared to that in the TIIA group. Treatment with TIIA reduced the expression of GFAP. **b**–**d** Expression levels of neuronal cell markers in the BMSCs + TIIA group were higher than those in the SCI group. The levels of NeuN in the BMSCs + TIIA group were the highest compared to those in the other groups. Values are the means ± SDs (*n *= 3 per group). *P < 0.05, **P < 0.01, ***P < 0.001 versus the SCI group. **e** The levels of GFAP were highest in the SCI group, while the levels of Nestin, NeuN, and NF200 were highest in the BMSCs + TIIA group compared to those in the other groups. Values are the means ± SDs (*n *= 3 per group). *P < 0.05, **P < 0.01 versus the SCI group

The levels of GFAP and neuronal cell marker proteins were examined at 28 days post-surgery using western blotting. As shown in Fig. 6a–d, the protein levels of GFAP in the SCI group were markedly higher than those in the other three groups (*n *= 3, *P < 0.05). In contrast, the protein levels of GFAP were significantly reduced in the BMSCs + TIIA group, while the protein levels of Nestin, NeuN, and NF200 were significantly higher, compared to those in the SCI group (*n *= 3, *P < 0.05, **P < 0.01, ***P < 0.001). These results indicated that combinational therapy with TIIA and BMSCs resulted in the production of a greater proportion of neuronal-like cells, and thus, the combination therapy is a more effective treatment for SCI compared to monotherapy.

### Real-time PCR for glial and neuronal gene expressions

GFAP, Nestin, NeuN, and NF200 gene expression were examined using real-time PCR on tissue harvested at 28 days post-surgery (Fig. 6e). The expression of GFAP in the SCI group was higher relative to that in the other three groups (*n *= 3, *P < 0.05), while the expression levels of Nestin, NeuN, and NF200 were significantly greater in the BMSCs + TIIA group compared to those in the SCI group (*n *= 3, *P < 0.05, **P < 0.01). Moreover, the expression of GFAP in the BMSCs + TIIA group was significantly lower than that observed in the SCI group (*n *= 3, **P < 0.01).

### Recovery of locomotor function

**Fig. 7:**
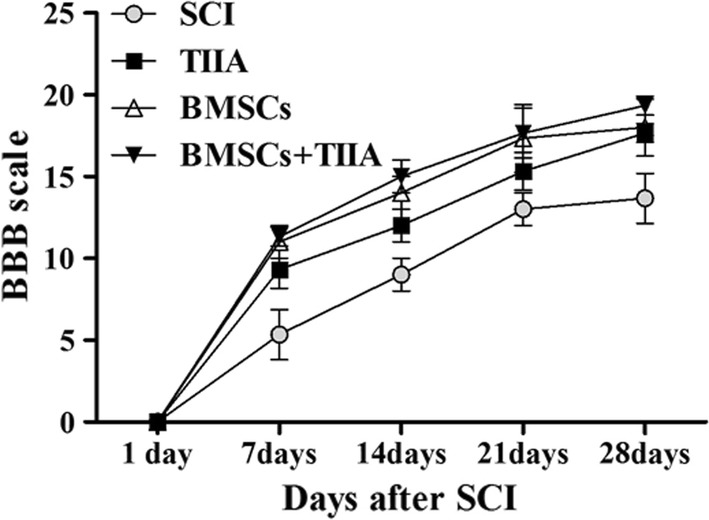
Recovery of locomotor function. Open field locomotor assessment using the Basso, Beattie, Bresnahan scale in each group at 1, 7, 14 21, and 28 days post-surgery. At the first day after injury, all of the rats had paralysis of hind limbs with partial recovery in the following weeks. Statistical analysis showed that the BBB scores of the rats in the three treatment groups were higher than those of the rats in the SCI group (*n *= 12 for each group, *P *< 0.05). There was no significant difference between these three groups (*P *> 0.05)

All SCI rats manifested complete hindlimb paraplegia at 1 day post-surgery (0 score) with partial recovery occurring during the following weeks. As shown in Fig. 7, the rats in the BMSCs + TIIA group improved more rapidly than those in the other groups. By 28 days post-SCI, the mean BBB values for the SCI, TIIA, BMSCs, BMSCs + TIIA groups were 13.67 ± 1.53, 17.67 ± 0.58, 18.00 ± 1.73, and 19.33 ± 0.58, respectively (Fig. 7). These behavioral results suggested that compared to SCI group, the other three groups are significantly higher (*P *< 0.05). However, there are no significant differences among the three treatment groups.

## Discussion

In SCI studies, there are in generally three main classes of rodent injury models: transection, contusion and compression. However, most humans suffer from an acute impact injury followed by a period of compression sometime later in their life. Therefore, many researchers use the ‘dura-intact’ compression and contusion injury model [29]. To simulate the events of SCI in this study, we used a modified aneurysm clip to squeeze the spinal cord without damaging the dura [30] and then examined the effects of TIIA’s treatment on BMSCs engraftment and differentiation.

Assessment of the SCI in our model entailed examining the extent of bilateral hindlimb movement and was considered successful if the rat’s hindlimbs were totally paralytic based on the BBB scale [31]. This scale was also used to assess locomotor outcomes post-treatment since this scale is sensitive and very reliable. Further, we assessed successful SCI by examining gross anatomical features, as well as cellular features via electron microscopy and H&E staining, which revealed edema and loose tissue structures in our model [26]. Trauma to the spinal cord leads to immediate mechanical damage and a cascade of secondary damage [32]. The secondary damage develops within from minutes to hours following initial mechanical damage, including the infiltration of inflammatory cells, the destruction of neuronal and glial cells, neuronal death, oxidative stress and locomotor dysfunction. In our study, there were no notably differences in secondary damage among the different groups.

To investigate the migration and proliferation of transplanted BMSCs into the lesioned spinal cord, engrafted BMSCs were prelabeled with a fluorescent membrane-intercalating dye, PKH67. Previous reports have shown that this fluorescent dye is ideal for in vivo cell tracking and cell proliferation studies [33]. In this current study, PKH67-labeled BMSCs migrated toward the lesion in the spinal cord by 28 days post-BMSCs transplantation with very few labeled cells detected in other regions (image not presented). This demonstrated that transplanted BMSCs by an intravenous injection were effectively delivered to the lesion without dispersion to uninjured sections.

Although transplanted BMSCs can migrate to the site of injury, BMSCs are capable of differentiating into mature neurons or glial cells [34]. The present study confirmed that the engrafted BMSCs could be induced to differentiate into neuronal-like and glial-like cells. Despite the ability of astrocytes to secrete neurotrophic factors and limit the pervasion of inflammatory reactions, extensive glial scarring within the lesioned area is inhibitory to axonal regrowth [35] and thus can perturb repair and behavioral outcomes. Neuronal-like cells can presumably replace missing or damaged neurons, modulate immune responses and facilitate tissue regeneration, thus improving the neurological deficits following an SCI.

To assess BMSC differentiation into glial-like cells, GFAP was used as an astrocytic marker. GFAP is an intermediate filament protein primarily found in astroglia that is often used as a marker for the activation of astrocytes following an injury such as SCI. As noted above, such activated astrocytes can lead to scar formation which is believed to inhibit neural regeneration [36]. In this study, we examined whether the combined treatment of BMSCs with TIIA could potentially improve SCI outcomes by attenuating of BMSC differentiation into astrocytes. The rationale for this is based on the observations that not only can TIIA significantly promote BMSCs migration and recruitment by the up-regulation of CXCR4 in a myocardial ischemia model [16, 17] but also that in the SCI model, TIIA administration can effectively attenuate the inflammatory response, apparently resulting in a decreased number of activated astrocytes and decreased glial scar formation [19]. Our data support these findings since TIIA treatment decreased the number PKH67-labeled cells that exhibited GFAP colocalization, and the protein level and gene expression of GFAP were decreased, as was glial scar formation. The present study reported a novel approach to optimize BMSC engraftment efficiency and increase its therapeutic effects based on the combination with TIIA in an SCI model. TIIA appears to improve BMSC engraftment and favor the differentiation of BMSCs into non-astrocytic cells.

To examine whether TIIA affected BMSC differentiation into neuronal cells, we examined the expression of three neuronal markers in PKH67-labeled engrafted BMSCs: Nestin, NeuN and NF200. Nestin is a type VI intermediate filament protein which is found in neural stem cells and can also be expressed in bone marrow stromal cells [37]. NeuN is observed in most neuronal cell types throughout the nervous system and first appears at a developmental time point that corresponds to the initiation of neuronal terminal differentiation [38]. NF200 is a 200-kDa neurofilament protein that appears to be an axonal marker [39]. According to many previous reports, these three proteins are all markers of neuronal differentiation and neural regeneration and play important roles in injured spinal cord tissue, so we used them to detect the effect of TIIA on the migration and differentiation of BMSCs. In our study, we found that the BMSCs + TIIA group exhibited more PKH67-labeled cells colocalized with neuronal markers by immunohistochemistry; higher expression levels of neuronal markers versus those in the other groups by western blot and real-time PCR at 28 days post-treatment; and Nestin+, NeuN+, or NF200+ cells preferentially located at the site of the lesion. According to prior reports, TIIA can also rescue impaired neurons, enhance neuronal regeneration [40] and attenuate edema and blood–brain barrier disruption [41]. Therefore, greater differentiation of BMSCs, more neural regeneration and less scar formation in the BMSCs + TIIA group in this study demonstrated that combination therapy of TIIA and BMSCs could promote BMSCs migration to the lesion and differentiation to neuronal-like cells. We also assessed the behavioral outcome of TIIA and BMSCs treatment in the SCI model. Based on the BBB scale, although there were no significant differences among the TIIA, BMSC and BMSCs + TIIA groups, we observed a faster functional recovery in the combination group compared with that in the control group and that in the groups treated with TIIA or BMSCs alone. Collectively, we demonstrated that TIIA promoted the differentiation of BMSCs into neurocyte-like cells in SCI rats and that this is concomitant with improved behavioral outcome.

One limitation of our current study is that we demonstrated this phenomenon without explaining the underlying mechanism(s). We speculate that TIIA may increase survival rates of transplanted BMSCs, increase the levels of trophic factors that BMSCs secrete into the lesion area, and reduce inflammatory cytokines. However, in order to understand the detailed mechanisms of the beneficial effects of TIIA, further experiments examining whether the combination of TIIA and BMSCs can also modulate secondary damage is required.

## Conclusions

The present study demonstrated that TIIA could promote the differentiation of transplanted BMSCs into neurocyte-like cells in an SCI model. Thus, TIIA treatment in combination with BMSCs transplantation may be a new therapeutic approach for SCI in clinical practice.

## Abbreviations

SCI: spinal cord injury
CNS: central nervous system
BMSCs: bone marrow mesenchymal stem cells
TIIA: tanshinone IIA
SMB: Salvia Miltiorrhiza Bunge
GFAP: glial fibrillary acid protein
NF200: neurofilament protein 200
SD: Sprague-Dawley
BBB: Basso, Beattie, Bresnahan
H&E: hematoxylin and eosin
DMEM/F12: Dulbecco’s modified Eagle’s medium F12
FBS: heat-inactivated fetal bovine serum
PE: phycoerythrin
APC: allophycocyanin
FITC: fluorescein isothiocyanate
ABC: avidin–biotin-complex
DAB: 3,3′-diaminobenzidine
RIPA: radioimmunoprecipitation assay
SDS: sodium dodecyl sulfate
PVDF: polyvinylidene fluoride
ECL: enhanced chemiluminescence
